# Exosomes: roles and therapeutic potential in osteoarthritis

**DOI:** 10.1038/s41413-020-0100-9

**Published:** 2020-06-19

**Authors:** Zhenhong Ni, Siru Zhou, Song Li, Liang Kuang, Hangang Chen, Xiaoqing Luo, Junjie Ouyang, Mei He, Xiaolan Du, Lin Chen

**Affiliations:** 1 Department of Wound Repair and Rehabilitation Medicine, Center of Bone Metabolism and Repair, Laboratory for Prevention and Rehabilitation of Training Injuries, State Key Laboratory of Trauma, Burns and Combined Injury, Trauma Center, Research Institute of Surgery, Daping Hospital, Army Medical University, Chongqing, China; 2 State Key Laboratory of Trauma, Burns and Combined Injury; Medical Cformation of H-type vessel in subchondral enter of Trauma and War Injury; Daping Hospital, Army Medical University of PLA, Chongqing, China; 3 Eleven Squadron Three Brigade, School of Basic Medical Science, Army Medical University, Chongqing, China

**Keywords:** Bone, Pathogenesis

## Abstract

Exosomes participate in many physiological and pathological processes by regulating cell–cell communication, which are involved in numerous diseases, including osteoarthritis (OA). Exosomes are detectable in the human articular cavity and were observed to change with OA progression. Several joint cells, including chondrocytes, synovial fibroblasts, osteoblasts, and tenocytes, can produce and secrete exosomes that influence the biological effects of targeted cells. In addition, exosomes from stem cells can protect the OA joint from damage by promoting cartilage repair, inhibiting synovitis, and mediating subchondral bone remodeling. This review summarizes the roles and therapeutic potential of exosomes in OA and discusses the perspectives and challenges related to exosome-based treatment for OA patients in the future.

## Introduction

Osteoarthritis (OA) is a highly prevalent type of degenerative joint disease that affects over 300 million people worldwide.^1^ Chronic pain and motion dysfunction induced by OA seriously reduced the quality of life of patients. In addition, the socioeconomic burden of OA on patients and society is considerable. Current OA management is broadly divided into nonpharmacological, pharmacological, and surgical treatments.^2–4^ Nonpharmacological treatments, such as exercise, weight loss, and physical therapy, are suggested as the appropriate therapy for early-stage OA patients. Pharmacological treatments are mainly aimed at achieving pain control for better function and quality of daily life. Surgical treatment is most widely used for end-stage patients with serious functional disability. At present, there are few satisfactory strategies to improve joint homeostasis and delay OA progression.^3,5^ Understanding the underlying mechanisms of OA can facilitate the development of novel therapies for future clinical needs.

OA has been previously described primarily in terms of articular cartilage destruction, but accumulating evidence has revealed that OA is a disease with whole-joint damage and dysfunction.^6,7^ During OA progression, the pathologic changes in joints include cartilage damage, remodeling of the subchondral bone, inflammatory activation in the synovium, degeneration of ligaments and the menisci, and changes in the joint capsule, bursa, periarticular muscles, nerves, and local fat pads. Several factors have been revealed to be associated with pathological changes in the OA joint, including aging, trauma, mechanical loading, and genetic and metabolic disorders.^4,8^ Moreover, the different tissues in the joint could influence each other during the course of OA, which may synergistically contribute to OA pathology and clinical symptoms.^9–11^ Subchondral bone is a layer of cortical bone below the articular cartilage and the underlying trabecular bone in the joint, which was recently proposed to play a significant role in OA pathogenesis. The subchondral bone could affect cartilage degeneration through mechanical changes or paracrine-mediated bone-cartilage cross-talk.^12–14^ The cytokines from synovial fibroblasts (SFB) of inflammatory cells could influence the degradation of the cartilage matrix and the formation of osteophytes by releasing proinflammatory factors such as IL-1β and bone-regulated factors including BMP-2.^15^ Inflammatory activation of the synovium and infrapatellar fat pad (IPFP) can lead to the release of various proinflammatory mediators that not only cause widespread changes in the structure and function of synovial tissue but also promote articular cartilage damage and accelerate OA development.^15–17^ Therefore, investigating intercellular communication within and/or among different joint cells during OA development could be beneficial for understanding the pathogenesis of OA and exploring new therapeutic strategies for OA in the future.

Exosomes are considered important mediators of cell–cell communication that participate in numerous physiological and pathological processes. Recently, the roles and therapeutic potential of exosomes in OA have been increasingly addressed in this field. In this review, we summarize the existing research on exosomes in OA and discuss the perspective and challenges related to exosome-based treatment for OA patients.

## Exosome

### Intercellular communication mediator

Extracellular vesicles (EVs) are membrane-bound vehicles that can be divided into three types, including exosomes, microvesicles (MVs), and apoptotic bodies.^18^ As an important kind of EV, exosomes have received the most attention over the past decade. Exosomes can be secreted by various cells and mediate intercellular communication via their contents, including lipids, nucleic acids, and proteins.

The diameter of exosomes usually ranges from 30–150 nm, and the density is between 1.13 and 1.19 g·mL^−1^.^19^ Trams et al. found that exfoliated membrane vesicles may serve a physiologic function and suggested these vesicles as exosomes.^20^ In 1983, Harding et al. observed that membrane-bound vesicles could be released by multivesicular endosome (MVE) exocytosis.^21^ Later, researchers found that the transferrin receptor could transfer from the surface of the cell into internal vesicles to form MVEs.^22^ In 1987, Johnstone et al. observed that exosome release during reticulocyte maturation was associated with plasma membrane activities.^23^ Raposo et al. later found that exosomes played an important role in antigen presentation and T cell activation.^24^ Then, the relationship between exosomes and tumors was reported.^25,26^ In 2007, Valadi et al. found that mRNA and microRNA can be sent to other cells by exosomes, indicating that exosomes may mediate intercellular communication by delivering nucleic acids.^27^ Thereafter, an increasing number of studies have shown that exosomes play important physiological and pathological roles by mediating cell–cell communication.^28,29^

#### Exosome biogenesis

**Fig. 1:**
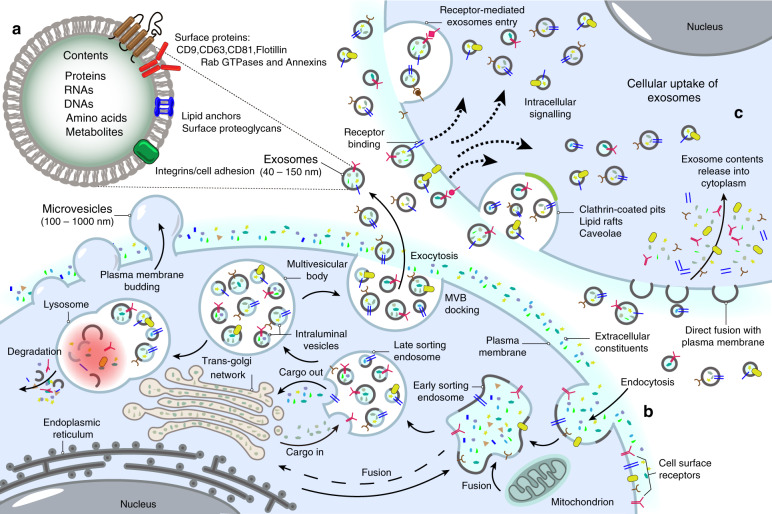
Biogenesis of exosomes. **a** Exosomes contain different types of proteins, nucleic acids, amino acids, and metabolites, in which CD9, CD63, CD81, flotillin, and Annexins could be used as markers. **b** Extracellular constituents along with cell surface proteins enter cells via the manners of endocytosis and plasma membrane invagination. Plasma membrane bud formation in the luminal side and the fusion of the bud with the constituents of the endoplasmic reticulum (ER), trans-Golgi network (TGN), and mitochondria lead to the formation of early sorting endosomes (ESEs). Then, ESEs give rise to late sorting endosomes (LSEs) in which second invagination via modification of the cargo, leading to the generation of various intraluminal vesicles (ILVs) and the formation of multivesicular body (MVBs). Next, some of MVBs fuse with lysosomes, and the contents in MVBs undergo degradation. Other MVBs can be transported to the plasma membrane and dock on the luminal side of cells. Finally, the exocytosis of MVBs releases ILVs as exosomes to the outside of cells. **c** Exosomes enter cells by different manners including fusion with cell plasma membranes, receptor-mediated entry, clathrin-coated pits, lipid rafts and so on

Exosome biogenesis can be divided into different phases, including early endosome formation by invagination of the plasma membrane, late endosome formation by cargo selection, the formation of multivesicular bodies (MVBs) from late endosomes and membrane fusion between MVBs and the plasma membrane, leading to the release of the vesicular contents, named exosomes^30–32^ (Fig. 1). The endosomal sorting complex required for transport (ESCRT), gene-2-interacting protein X linked by apoptosis and tumor susceptibility gene 101 (TSG-101), is mainly responsible for the formation and release of exosomes.^33–36^ The ESCRT is mainly composed of ESCRT-0, ESCRT-I, ESCRT-II, and ESCRT-III in complex.^37^ ESCRT-0, -I and -II contain ubiquitin-binding subunits that can capture ubiquitin-tagged cargo, while ESCRT-III contributes to vesicle budding and scission.^38,39^ However, exosome formation can occur independently of the ESCRT complex under certain conditions. The lipids are capable of promoting exosome formation. For example, the transfer of exosome-associated domains into the endosome lumen in a ceramide-activated model does not rely on ESCRT function.^39,40^

#### Methods of exosome separation

The enrichment of exosomes with high purity was the critical step for basic research and the further clinical application of exosomes. Currently, different methods for exosome separation, including ultracentrifugation, size-based techniques, immunoaffinity purification, precipitation, and microfluidics-based isolation techniques, have been developed based on the size, shape, density, and surface proteins of exosomes. These methods have their own advantages and disadvantages and need to be further improved to promote the research on exosomes and their application.^41–45^

##### Ultracentrifugation

Ultracentrifugation is the most common method to separate different biological components, such as viruses, bacteria, subcellular organelles, and EVs.^43^ Ultracentrifugation is currently considered the classical method of exosome isolation and can be divided into density gradient ultracentrifugation and differential ultracentrifugation. A centrifugal force between ×100 000 and 150 000 g is often used in this separation method. However, ultracentrifugation has some shortcomings, such as requiring advanced supercentrifugation and consuming extensive time. Moreover, ultracentrifugation may influence the structures of exosomes, which would impede downstream analysis.

##### Size-based technique

Size-based technique such as ultrafiltration is another kind of isolation method and has been used to harvest exosomes from urine, serum, cerebrospinal fluid, and cell culture medium by particle size or molecular weight.^42,46,47^ This exosome isolation method does not require complex equipment, and the operation procedure is relatively simple. However, this method still poses some challenges.^42,43^ For example, the isolated exosome may be contaminated by molecular aggregates, decreasing exosome purity. In addition, the shear stress produced in the isolation process may induce the deterioration of exosomes.

##### Immunoaffinity purification

Based on marker proteins in the bilayer membranes of exosomes, exosomes can be selectively separated through immunoprecipitation mediated by specific immobilized antibodies.^42^ This isolation method could effectively improve exosome purity in some cases without complicated procedures. In addition, immunoaffinity purification could distinguish different subgroups of exosomes according to their special marker proteins, which may be beneficial for detailed mechanistic investigations in the future. Nevertheless, this method requires excellent antibody specificity, rigorous sample preparation, and costly reagents. In addition, only those EVs expressing the antibody-recognized protein can be separated, which may result in a low yield of exosomes.^48^

##### Precipitation

Precipitation with polyethylene glycols (PEGs) is widely used to isolate viruses and small particles.^43,49–51^ Polymers can adhere to water molecules, decreasing exosome solubility, and this effect can be used to separate exosomes from conditioned media, serum, or urine.^48^ The use of precipitation for exosome isolation is convenient and does not require special equipment. In addition, the concentration of isolated exosomes is relatively high. However, there are still many problems with this method, such as low recovery and high impurities.

##### Microfluidics-based isolation techniques

Microfluidics-based isolation is an alternative isolation method that is based on physical and biochemical properties, such as size, density, and immune affinity. In addition, it is a new sorting method that involves acoustic, electrophoretic, and electromagnetic procedures.^52–54^ The steps of this method mainly include immunoaffinity, sieving, and trapping exosomes on porous structures.^55^ This method consumes small amounts of sample volume, reagents, and separation time.^56^ In addition, microfluidics-based isolation could synergistically enrich exosomes and improve purity in combination with other exosome separation methods.^43^ However, this method requires advanced equipment, which may restrict its large-scale application.

#### Exosome identification

Exosome identification is mainly based on morphological features, particle size, and signature proteins such as CD9, CD63, CD81, and HSP90.^57^ There are different methods to identify the characteristics of exosomes.^43^ First, scanning electron microscopy (SEM) or transmission electron microscopy (TEM) can be used to identify exosomes directly. SEM observes the exosome surface microstructure, while TEM has a maximal resolution of 0.2 nm and can reveal the internal structure and morphology of exosomes.^58^ Second, nanoparticle tracking analysis (NTA) could analyze the particle size and the concentration of exosomes. The process of NAT-based detection is relatively simple, and the result can be better quantified. Third, western blot technology contributes to estimating specific marker proteins in exosomes, including CD63, CD8, TSG101, flotillin-1, ALIX, CD9, CD81, and CD82. Fourth, flow cytometry (FCM) can be used to analyze the size of exosomes by labeling targeted exosomes with specific antibodies or fluorescent dyes. FCM has some advantages for exosome analysis, including high-throughput screening and data quantification. Moreover, FCM can be used to distinguish different subpopulations of exosomes. In addition to the above methods, atomic force microscopy, tunable resistive pulse sensing, and dynamic light scattering (DLS) could also be used for the identification of exosomes.^43^

#### The function of exosomes in bone homeostasis

Over the past decades, exosomes have been found to affect several physiological and pathological processes via the exosomal contents, including RNAs, DNAs, proteins, and lipids.^59^ To date, cumulative evidence has revealed that exosomes participate in many biological processes, including angiogenesis, cell differentiation, immunomodulation, metabolic balance, and development,^24,26,60–62^ and are highly involved in many diseases, such as cancer, neurodegenerative disease, autoimmune diseases, and cardiovascular diseases.^63–73^ Recently, the roles of exosomes in bone homeostasis have been extensively addressed.^73^

Bone homeostasis is primarily maintained by bone resorption and bone formation, which are involved in various types of cells, including osteoblasts, osteoclasts, osteocytes, and MSCs.^74^ Abnormalities in bone homeostasis are closely related to bone diseases in several ways, such as affecting the onset and progression of osteoporosis and the wound repair of bone fractures. Many studies have shown that cells in the bone microenvironment can secrete exosomes to regulate bone resorption and bone formation. Osteoblast-derived exosomes carry potential osteogenesis-related signaling, such as the eukaryotic initiation factor-2 pathway, which may participate in bone formation.^75^ Exosomes from mineralizing osteoblasts could promote the differentiation of bone marrow stromal cells to osteoblasts.^76^ Exosomes from osteoclasts could inhibit osteoblast function, osteogenic differentiation, and bone formation via exosomal miRNAs, including miR-214-3p and miR-23a-5p.^77–79^ miR-218 contained in osteocyte-derived exosomes plays an important role in the myostatin-mediated inhibition of osteoblastic differentiation.^80^ In addition, MSCs can produce and release exosomes to participate in maintaining bone homeostasis. The exosomes produced by MSCs can prevent osteocytes from undergoing apoptosis in a hypoxia/serum deprivation model and glucocorticoid-induced osteonecrosis model.^81,82^ The exosomes excreted by iPS-derived MSCs can promote the regeneration of bone defects via enhanced angiogenesis and osteogenesis in an ovariectomized rat model.^83^ Exosomal transfer RNA-derived fragments from the plasma of osteoporosis patients, including tRF-25, tRF-38, and tRF-18, were significantly increased compared with those from healthy controls, suggesting that these tRFs may be new diagnostic biomarkers for osteoporosis.^84^ Exosomes from the BMSCs of osteoporosis patients decrease the osteogenic ability of MSCs by targeting the microRNA-21/SMAD7 pathway.^85^ Exosomes from different tissue-derived MSCs or iPS cell-derived MSCs could prevent bone loss, promote bone formation, and prevent osteoporosis in experimental animal models,^86–89^ indicating that MSC-derived exosomes could be a potential therapeutic treatment for osteoporosis.^90^

In addition to osteoporosis, MSC-derived exosomes also greatly contribute to fracture healing.^91^ The exosomes from hypoxically preconditioned MSCs had a higher miR-126 level and promoted fracture healing via the SPRED1/Ras/Erk signaling pathway.^92^ The injection of BMSC-derived exosomes can rescue the retardation of fracture healing in the femur fracture model of CD9(−/−) mice.^93^ Similarly, BMSC-derived exosomes can enhance osteogenesis and angiogenesis partially through the BMP-2/Smad1/RUNX2 and HIF-1alpha/VEGF pathways at fracture sites to accelerate fracture repair in a femoral nonunion rat model.^94^ Moreover, umbilical cord MSC-derived exosomes also promote fracture healing in a rat model of stabilized fracture, which is involved in the HIF-1alpha-mediated regulation of angiogenesis.^95^

In addition to osteoporosis and fracture, an increasing number of studies have revealed that exosomes are closely involved in OA pathology and have strong therapeutic potential for this disease.

### Exosomes in OA

**Fig. 2:**
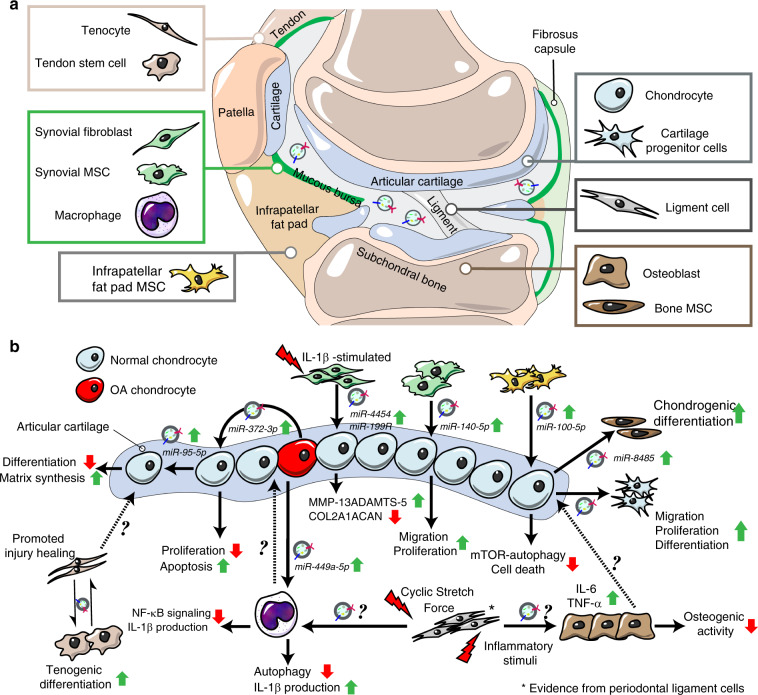
The exosomes from different tissues of OA joint and their potential biological effects. **a** The exosomes can be detected in the articular cavity and changed during OA progression. Some studies showed that the joint cells including chondrocytes, osteoblasts of subchondral bone, synovial mesenchymal stem cells (MSCs) and fibroblasts, infrapatellar fat pad MSCs, tenocytes and tendon stem cells as well as periodontal ligament cells and stem cells produce and release exosomes, which may be involved in the regulation of joint homeostasis. **b** The exosomes derived from different joint cells could mediate cell–cell communications and regulate diverse cell phenotype including cell proliferation, migration, differentiation, autophagy, matrix synthesis, inflammatory reaction and etc

**Fig. 3:**
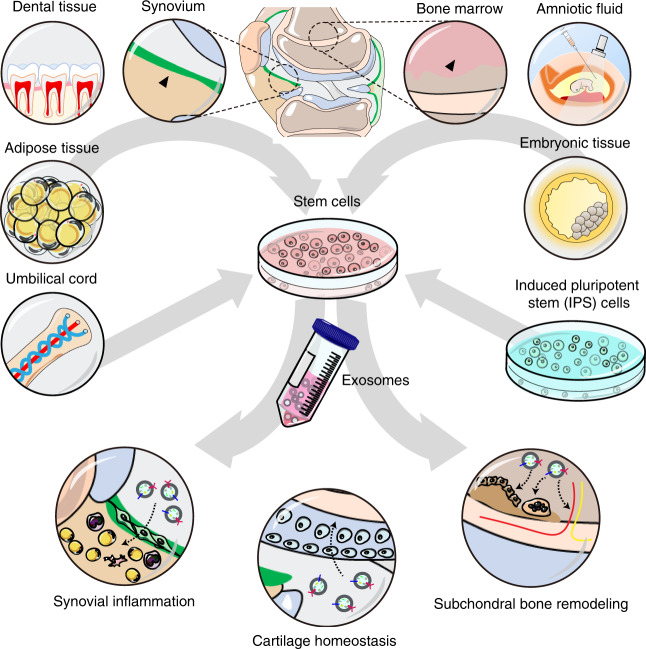
The therapeutic effects of stem cells-derived exosomes on OA. The exosomes from different tissues-derived stem cells or iPS-derived MSCs protect joints from OA via regulating synovial inflammation, cartilage homeostasis, and subchondral bone remodeling

The roles and underlying mechanisms of exosomes in OA have attracted great interest from researchers. To date, there are two main research directions in this field. One set of studies have mainly focused on the diagnostic significance and biological effects of endogenous exosomes during OA (Fig. 2). In another, researchers have paid attention to the therapeutic effects of stem cell-derived exosomes on OA and potential optimization strategies (Fig. 3).

#### Exosomes from joint tissues

Some research has indicated that exosomes are present in human synovial fluid (SF).^96–99^ Moreover, the quantity and composition of SF-derived exosomes could be changed in joint diseases, including OA, rheumatoid arthritis, and reactive arthritis.^99–101^ Skriner et al. reported that citrullinated proteins, including fibrinogen D fragment, Sp alpha (like CD5 antigen protein) receptor, fibrinogen beta-chain precursor, Sp alpha (like CD5 antigen protein) receptor, fibrin beta-chain, and fibrin alpha-chain fragment, were observed in synovial exosomes from RA patients but not in those with OA, indicating that exosomes carrying citrullinated peptides may be unique in different joint diseases.^97^ Kolhe et al. revealed the characterization of miRNAs in exosomes from the SF of OA patients. Their data showed that the SF-derived exosomal miRNAs differed between male and female OA patients and that the specific miRNAs for female OA patients are relevant to the estrogen response and toll-like receptor signal.^99^ OA SF-derived EVs functionally influence the expression of anabolic and catabolic activity-related genes.^99^ In addition, Domenis et al. found that exosomes from the SF of patients with gonarthrosis function to enhance the production of proinflammatory factors from M1 macrophages, which suggests that SF-derived exosomes may play a role in the pathological process of joint diseases.^96^

Zhao et al. reported that SF samples obtained from both early OA and late-stage OA contained much higher exosome levels than those from controls.^101^ In addition, the levels of exosomal lncRNA PCGEM1 from OA SF gradually increased with OA progression.^101^ Moreover, Gao, K. demonstrated that exosomes from end-stage knee OA patients have a higher level of chemokines and could recruit inflammatory cells and inhibit cartilage proliferation, which may ultimately promote joint degeneration.^98^ The above data provide the clinical relevance of exosomes and OA pathology, which improves our understanding of the OA microenvironment from the new perspective of exosomes (Table 1).

**Table 1:** The exosomes from different joint cells and their biological actions on the target cells

Tissue	Cell type	Separation method	Exosome diameter	Target cells	Biological effects	Mechanisms of actions
Cartilage	Human OA chondrocytes	Precipitation	–	Chondrocytes	Inhibit chondrocytes proliferation and promote the apoptosis of chondrocytes.	GSK-3β-mediated regulation of HULC and miR-372-3p in exosomes.^110^
Cartilage	Mouse primary chondrocytes	Ultracentrifugation	40–110 nm	Chondrocytes and macrophages	Restore mitochondrial dysfunction and polarize macrophage response toward an M2 phenotype.	The restoration of normal mitochondrial structure and moderate ROS production.^106^
Cartilage	Human OA chondrocytes	Ultracentrifugation and Size-based filtration	30–150 nm	Macrophages	Stimulate inflammasome activation and increase IL-1β production.	Inhibit ATG4B expression via miR-449a-5p, which leading to inhibition of autophagy in LPS-primed macrophages.^107^
Cartilage	Rabbit articular chondrocytes	Size-based filtration	30–200 nm	Chondrocyte-progenitor cells (CPCs)	Promotes ectopic chondrogenesis and inhibiting angiogenesis.	Stimulate CPCs proliferation and increase the expression of chondrogenesis markers.^108^
Cartilage	Chondrocytes (cell line)	Ultracentrifugation		Bone marrow mesenchymal stem cells (BMSCs)	Promote chondrogenic differentiation of BMSCs	Activate Wnt/b-catenin pathways via targeting GSK-3b by exosomal miR-8485.^111^
Cartilage	MiR‐95‐5p‐overexpressing chondrocytes	Ultracentrifugation	90–150 nm	Chondrocytes	Regulate cartilage development in hMSCs during chondrogenesis and promote cartilage matrix expression.	Inhibit histone deacetylase 2/8 expression via MiR‐95‐5p.^109^
Synovial tissue	IL-1β-stimulated synovial fibroblasts	Ultracentrifugation and precipitation	40-100 nm	Articular chondrocytes	Induce OA-like changes both in vitro and in ex vivo models.	Upregulate MMP-13 and ADAMTS-5 expression and down-regulate COL2A1 and ACAN in chondrocytes.^121^
Synovial tissue	MiR-140-5p-overexpressing human synovial mesenchymal stem cells	Affinity-Based capture	30–150 nm	Articular chondrocytes	Enhance proliferation and migration of chondrocytes without decreasing ECM secretion in vitro Promote cartilage regeneration and maintain cartilage matrix content in vivo	Wnt5a and Wnt5b carried by exosomes to activate Wnt/YAP signaling pathway MiR-140-5p-mediated inhibition of RalA and increase of SOX9 and Aggrecan.^122^
Subchondral bone	Osteoblast cells	Ultracentrifugation	30–150 nm.^135^	–	–	–
Infrapatellar fat pad	Infrapatellar fat pad MSCs	Ultrafiltration, precipitation and affinity-based capture	30–150 nm	Articular chondrocyte	Chondroprotective effects and ameliorates gait abnormalities.	MiR100-5p-mediated inhibition of mTOR pathway.^146^
Tendon	Tendon stem cells	Ultracentrifugation	40–200 nm	Tendon stem cells	Promote healing of injured tendon through regulating the metabolism of the tendon extracellular matrix and increases the ultimate stress and maximum loading in tendon.	Decrease MMP‐3 expression, increase TIMP‐3 and Col1a1 expression.^150^
Tendon	Tenocyte	Ultracentrifugation	–	Mesenchymal stem cells	Promote MSCs to undergo the tenogenic differentiation.	TGF-β-dependent manner.^131^
Ligament	Periodontal ligament fibroblasts	Ultracentrifugation and precipitation	70–100 nm	MG-63 osteoblasts	Induce inflammation and inhibit osteogenic activity in osteoblasts	Upregulate the levels of IL-6 and TNF-α, inhibit OPG expression.^157^
Ligament	Periodontal ligament stem cells	Ultracentrifugation and affinity-based capture	119 ± 12.1 nm^155^	–	–	–
Ligament	Periodontal ligament cells	Precipitation	30–100 nm	Macrophage	Regulate macrophages function and maintain inflammation homeostasis.	Suppress IL-1β production via inhibiting NF-κB signaling pathway.^156^

##### Cartilage-derived exosomes

Chondrocytes are the only cell type in articular cartilage. Previous studies have revealed that chondrocytes can release small membrane-bound extracellular articular cartilage matrix vesicles (ACVs) with a diameter of 100 nM to participate in the pathologic mineralization of osteoarthritic articular cartilage.^102,103^ Exosomes have been shown to share many features of matrix vesicles, including lipid and protein content, size and morphology, suggesting that matrix vesicles and exosomes may be homologous.^104^ Moreover, these ACVs contain significant amounts of RNA, similar to exosomes, which are protected from enzymatic degeneration and can be transferred to intact primary chondrocytes.^105^ The distinction between ACVs and chondrocyte-derived exosomes is still unclear and needs more research. Nevertheless, an increasing number of studies have revealed that both ACVs and chondrocyte-derived exosomes could mediate cell–cell communication.^105–109^ Exosomes from primary chondrocytes cultured in a normal environment (D0 exosomes) could restore mitochondrial dysfunction and enhance M2 macrophage penetration with a reduction in M1 macrophages.^106^ The intra-articular administration of D0 exosomes efficiently delayed OA progression.^106^ We found that exosome-like vesicles derived from osteoarthritic chondrocytes could stimulate inflammasome activation and increase mature IL-1β production in macrophages via the miR-449a-5p/ATG4B/autophagy pathway, which may aggravate synovitis and accelerate the progression of OA.^107^ In addition, Chen et al. reported that exosomes derived from chondrocytes (CC-Exos) could induce efficient ectopic chondrogenesis of cartilage progenitor cell (CPC) constructs in subcutaneous environments, which may represent a cell-free therapeutic approach for cartilage regeneration.^108^ Moreover, chondrocyte-secreted exosomes could affect chondrogenic differentiation and cartilage matrix synthesis via the miR-95-5p-regulated expression of HDAC2/8.^109^ The exosomes from OA chondrocytes could inhibit cell proliferation and enhance the apoptosis of chondrocytes, which was involved in the GSK-3β-mediated enrichment of HULC and decrease in miR-372-3p in exosomes.^110^ In addition, exosomes from chondrocytes could promote the chondrogenic differentiation of BMSCs by activating the Wnt/β-catenin pathway, which was related to the inhibition of GSK-3β expression by exosomal miR-8485.^111^ These results supported that chondrocytes could produce exosomes that mediate cell–cell communication, which play a functional role in AC maintenance and OA pathogenesis.

##### Synovium-derived exosomes

The synovium is a key component of synovial joints and greatly contributes to maintaining the homeostasis of articular cartilage. Abnormalities of the synovium, especially low-grade inflammation, have been reported to be associated with clinical signs and joint histopathology of OA patients.^112–117^ The inflammatory and anti-inflammatory cytokines produced by synovial macrophages and synoviocytes have been found to play important roles in regulating cartilage degradation and osteophyte formation.^15,118–120^ However, the role and cross-talk between synovium-derived exosomes and joint cells in OA pathology are poorly understood.

Headland et al. reported that neutrophil-derived MVs can penetrate cartilage, indicating that EVs from the synovium could mediate the cross-talk between the synovium and cartilage.^100^ Kato et al. isolated exosomes from IL-1beta-motivated SFB of human normal knee joints and cocultured these exosomes with articular chondrocytes.^121^ They found that exosomes from IL-1beta-stimulated SFB dramatically increased the levels of catabolism-related genes, including MMP-13 and ADAMTS-5, while decreasing the expression of anabolism-related genes, including COL2A1 and ACAN.^121^ Moreover, the exosomes from IL-1beta motivated SFBs to induce more proteoglycan derived from cartilage explants.^121^ These data first suggested that exosomes from the synovium could induce OA-like changes in vitro and ex vivo.^121^ In addition, synovial mesenchymal stem cells (MSCs) could also produce functional exosomes (SMSC-Exos) that could be taken up by articular chondrocytes.^122^ These SMSC-Exos activated YAP via dephosphorylation and decreased ECM secretion, and they induced articular chondrocyte proliferation and migration via Wnt5a and Wnt5b signaling.^122^ Moreover, exosomes collected from miR-140-5p-overexpressing synovial MSCs (SMSC-140s) promoted the proliferation and migration of articular chondrocytes but did not affect the secretion of ECM in vitro.^122^ The SMSC-140s also significantly ameliorated the severity of joint wear in a rat OA model.^122^ These findings revealed that the synovium could release functional exosomes, and their function can be regulated by their contents. In addition to SFB and MSCs, exosomes from other synovial cells, including macrophages, T cells and endothelial cells, should also be given close attention. Recently, Tsuno et al. investigated the effects of salazosulfapyridine and methotrexate on the proteome of exosomes produced by a human synovial sarcoma cell line (SW982).^123^ Their data indicated that these anti-rheumatic drugs changed the protein profiles of SW982-derived exosomes and inhibited the effect of IL-1beta on the exosomal proteome.^123^ The effects of anti-OA strategies on synovium-derived exosomes are worth further study.

##### Subchondral bone-derived exosomes

Subchondral bone plays an important role in the protection of normal joints and undergoes structural changes, including bone sclerosis, during the OA process.^124,125^ The remodeling of subchondral bone was strongly associated with the grade of cartilage lesions in clinical OA patients^126^ and somewhat related to joint pain in some cases.^127^ In experimental OA models, targeting specific signals in subchondral bone, such as TGF-β, could attenuate the pathological severity of OA and reduce the pain response.^13,128–130^ Previous studies revealed that exosomes could function by regulating the TGF-β signal of their targeted cells.^131–133^ In addition, Cai et al. reported that exosomes from TGF-β1 gene-modified dendritic cells delayed the development of inflammatory bowel disease by increasing CD4 + Foxp3 + Tregs and inhibiting Th17 in a dextran sodium sulfate (DSS)-induced mouse model.^134^ The effects of exosomes on the signaling pathway of subchondral bone cells, including TGF-β signaling, and their potential roles in OA progression deserve further attention. Cells, including osteoblasts, osteoclasts, osteocytes and bone marrow MSCs, can secrete exosomes to regulate the bone microenvironment and mediate cell–cell communication,^76–82^ but their roles and underlying mechanisms in the remodeling of subchondral bone during the OA process are not well known. Recently, Liu et al. harvested subchondral bones from OA patients with different severities of joint wear and obtained osteoblasts from them.^135^ They determined that osteoblast cells from OA subchondral bones could produce exosome-like EVs 30~150 nm in diameter containing exosomal markers (CD9, HSP70, and Flotillin-1).^135^ Moreover, these exosomes from the osteoblast cells of subchondral bones (SB-OC-EXOs) from OA patients contained high levels of hsamiR-4717-5p, hsa-miR-885-3p, hsa-miR-135a-3p, hsamiR-210-5p, and hsa-miR-1225-5p.^135^ The physiological and pathological effects of SB-OC-EXOs need further investigation. In addition, exosomes from other cells of subchondral bones are also worthy of attention.

##### Infrapatellar fat pad-derived exosomes

Infrapatellar fat pad (IPFP), also termed Hoffa’s fat pad, is a knee adipose tissue that plays important roles in knee joint function and pathology.^136,137^ The size of the IPFP is closely related to knee cartilage volume and structural abnormalities in the clinic.^138^ In addition, an increasing number of studies have identified IPFP as an emerging source of inflammation that could contribute to OA progression, including pathological severity and knee pain.^136,137,139–141^ Moreover, IPFP-derived MSCs (IPFP-MSCs) presented potent capability for cartilage regeneration in vitro and in vivo, suggesting that these stem cells are promising cell sources for OA treatment.^142–145^ Recently, we separated and identified exosomes from IPFP-MSCs (MSC(IPFP)-Exos).^146^ Our data demonstrated that MSC(IPFP)-Exos can ameliorate gait abnormalities in OA mice and alleviate articular cartilage lesions in vivo.^146^ Exosomal RNA-seq revealed that miR-100-5p was highly abundant in MSC(IPFP)-Exos.^146^ MSC(IPFP)-Exos may regulate the biological behaviors of chondrocytes via miR-100-5p-mediated inhibition of mTOR signaling.^146^ In addition to exosomes from MSCs, the physiological and pathological effects of exosomes from other IPFP-derived cells, including adipocytes, macrophages, lymphocytes, and granulocytes, should also be investigated in the future.

##### Tendon-derived exosomes

The tendon is an important part of the joint structure that maintains joint stability and regulates the range of motion. Abnormalities of the tendon have been observed during OA progression and shown to be related to OA development.^147–149^ Ibrahim et al. found increased tendon degeneration in patients with shoulder OA.^147^ The samples from OA-derived tendons presented a degenerative appearance with increased scar tissue and noncollagenous ECM.^149^ Failed repair of the subscapularis tendon (SSC) significantly increased the risk of developing secondary glenohumeral OA,^148^ which indicates that enhancement of tendon repair may be a potential strategy for preventing secondary OA after joint injury. Recently, some studies revealed that tendon-derived exosomes could promote tendon repair and regeneration.^131,150^ Xu et al. reported that tenocytes could secrete exosomes that promote the tenogenic differentiation of MSCs.^131^ Moreover, the tenogenic differentiation of MSCs induced by tenocyte-derived exosomes could be blocked by the inhibition of TGF-beta signaling.^131^ In addition, Wang et al. harvested exosomes from conditioned culture medium of tendon stem cells (TSCs) using ultra-high-speed gradient centrifugation and estimated the effect of these exosomes on tendon injury healing.^150^ Their experimental results showed that TSC-derived exosomes significantly enhanced tendon matrix maintenance in vitro and increased the biomechanical characteristics of ultimate tension and maximal charging in a rat Achilles tendon tendinopathy model.^150^ More research is needed on role of tendon-derived exosomes in OA cartilage or other tissues.

##### Ligament-derived exosomes

The ligament is an important intraarticular structure that greatly contributes to joint function. A previous study revealed that the degeneration of the intraarticular ligament was highly associated with cartilage and bone damage during the OA process.^151^ Destruction of ligament integrity, such as the cruciate ligament, seriously decreased joint stability and promoted the development of knee OA, indicating an important role of the ligament in OA disease.^152–154^ Zhao et al. isolated exosomes from periodontal ligament stem cells using an ultracentrifugation method and identified their exosomal characteristics by TEM, western blot and nanosight tracing analysis.^155^ Cyclic stretch force promoted the secretion of exosomes from periodontal ligament cells (PDL cells), and these PDL-derived exosomes inhibited the production of IL-1beta in macrophages through the NF-kappaB signaling pathway.^156^ In addition, Zhao et al. also revealed that human PDL fibroblasts (hPDLFs) could produce exosomes.^157^ The hPDLF-derived exosomes promoted the inflammatory response and inhibited the osteogenic activity of osteoblasts, which affected bone remodeling in vitro.^157^ In addition to the PDL, exosomes from joint ligaments, such as the anterior cruciate ligament of the knee joint, should be further studied. Moreover, it is worth exploring the underlying mechanisms of ligament-derived exosomes in joint diseases, including OA.

#### The therapeutic effects of stem cell-derived exosomes on OA

Stem cells such as bone marrow mesenchymal stem cells (BMSCs) and adipose mesenchymal stem cells (AMSCs) have shown potent cartilage regeneration ability and have been clinically tested for OA treatment.^158–160^ Clinical trials of intra-articular injection of MSCs into the knee with OA have exhibited reliable safety and feasibility,^161,162^ while this approach could ameliorate the knee society clinical rating system (KSS) and OA outcome score^163,164^ and partially alleviate knee pain.^165–168^ However, the detailed mechanisms for this and other stem cell-based OA treatments have not been well clarified. An increasing number of studies have suggested that the therapeutic effects of stem cells are mainly dependent on the paracrine function of stem cells, including the secretion of EVs.^160,169,170^ To date, exosomes from different types of stem cells have been revealed to regulate cartilage regeneration and attenuate OA progression in certain models (Fig. 3, Table 2).

**Table 2:** The therapeutic effects and underlying mechanisms of exosomes derived from stem cells on OA

Exosomes	Separation method	Mechanisms of actions	Biological effects
BMSCs- derived exosomes	Ultracentrifugation and Ultrafiltration	• Prevent OA chondrocytes from apoptosis by p38, ERK, and akt signaling pathways.^182,183^ • Regulate catabolism and anabolism in chondrocytes.^182^ • Maintain mitochondrial membrane potential and inhibit mitochondrial dysfunction.^183,184^ • Suppress osteoclast activity in subchondral bone via RANKL-RANK-TRAF6 pathway.^185^ • Inhibit proliferation and enhance apoptosis in synovial fibroblasts via microRNA-26a-5p/ PTGS2 pathway.^186^	• Reduce the damage of articular cartilage.^182,185^ • Abrogate the degradation of subchondral bone.^182,185^ • Inhibit aberrant nerve invasion and abnormal formation of H-type vessel in subchondral bone.^185^ • Relieve pain in OA model.^185^ • Decrease the infiltration of inflammatory cells, down-regulate the level of inflammatory factor and alleviate pathological changes of synovium.^186^ • Inhibit the activation of macrophages.^182^
EMSCs- derived exosomes	Immunoaffinity purification and Ultracentrifugation	• Promote M2 macrophages infiltration, decrease M1 macrophages and proinflammatory cytokine production.^218^ • Activate adenosine-dependent AKT and ERK signaling pathways by exosomal CD73.^218^ • Reverse IL-1β-mediated inhibition of s-GAG synthesis and weaken the nitric oxide and MMP13 production via adenosine-mediated activation of AKT, ERK and AMPK.^220^ • Enhance miR-135b expression and decrease Sp1 expression.^221^	• Repair the damage of cartilage and subchondral bone.^217,220,221^ • Enhance surface regularity and integration with adjacent host cartilage.^218^ • Promote chondrogenic formation.^218^ • Regulate the migration, proliferation and matrix synthesis of chondrocytes.^218^ • Prevent from cartilage destruction and matrix degradation.^219^ • Suppress inflammation and restore matrix homeostasis.^220^
AMSCs- derived exosomes	Ultracentrifugation and Ultrafiltration	• Inhibit the activity of senescence-associated β-Galactosidase and γH2AX foci accumulation, reduced IL-6 and PGE2 levels, enhanced the release of IL-10.^208^ • Restrain the production of proinflammatory mediators TNF-α, IL-6, PGE2 and NO, and reduce the MMPs activity and MMP-13 expression, enhance the levels of the IL-10 and Collagen-II.^209^ • Promote chondrocytes viability, maintain the balance of anabolism and catabolism via miR-100-5p-mediated mTOR inhibition and autophagy enhancement.^146^ • Enhance proliferation and chondrogenic potential of periosteal cells via upregulating miR‑145 and miR‑221^211^	• Down-regulate mitochondrial membrane potential.^208^ • promote chondrogenesis in periosteal cells and increase chondrogenic markers^211^ • Show the potential anti-inflammatory and chondro-protective effects.^208,209^ • Ameliorate the pathological severity of articular cartilage and partially improve the abnormal gait.^146^
SMSCs- derived exosomes	Ultrafiltration	• Promote the proliferation and migration of chondrocytes via activation of YAP, prevent the ECM from damage through miR-140-5p/RalA-mediated increase of SOX9 and Aggrecan in vitro.^122,200^ • Reverse GC-induced proliferation inhibition and apoptosis of BMSCs.^201^	• Promote cartilage regeneration, Maintain the content of collagen II and attenuate OA progression.^122,195,200^ • Decrease the glucocorticoid (GC)-induced trabecular bones loss, bone marrow necrosis and fatty cells accumulation, improve the bone mineral density and the microstructures of the trabecular bone.^201^
AFSCs- derived exosomes	Precipitation	• Inhibit M1 polarization, decrease the expression of CD86, iNOS and IL-1 R1, regulate immunosuppressive and chondrogenesis via exosomal TGFβ and IDO.^222^	• Enhance pain tolerance level and induce an almost complete restoration of hyaline cartilage with good surface regularity.^222^
SHEDs- derived exosomes	Ultracentrifugation	• Inhibit miR-100-5p-mediated mTOR expression.^224^	• Inhibit inflammatory reaction and maintain anabolism homeostasis of chondrocytes.^224^
iMSCs- derived exosomes	Ultrafiltration	• Promote chondrocytes migration and proliferation.^200^	• Decrease the OARSI in experimental OA model, present a potent therapeutic effect on OA.^200^

##### Exosomes from bone mesenchymal stem cells

The exosomes derived from BMSCs could affect cell fate, including apoptosis, proliferation, invasion, and migration.^171,172^ Moreover, BMSC-derived exosomes can regulate a variety of physiological and pathological processes, including the immune response, osteogenesis, fibrosis, and angiogenesis.^94,173–175^ Several studies have reported that BMSC-derived exosomes significantly promote the regeneration and repair of injured tissues, including cartilage and subchondral bone.^174,176–181^ The exosomes as well as MVs/microparticles (MPs) from TGFβ3-pretreated BMSCs significantly increased the expression of anabolic markers and decreased the levels of catabolic marker genes in osteoarthritic chondrocytes.^182^ In addition, these BMSC-derived exosomes could prevent osteoarthritic chondrocytes from undergoing apoptosis.^182^ Qi et al. observed that BMSC exosomes could be taken up by chondrocytes to abolish IL-1β-induced cell apoptosis and damage to the mitochondrial membrane potential, in which p38, ERK, and Akt pathways were involved.^183^ Chen et al. also reported that chondrocytes could take up BMSC exosomes labeled with Dio fluorescent dye.^184^ These Dio-labeled exosomes were mainly localized in the perinuclear region of chondrocytes and could be fused with the mitochondria.^184^ Their data also suggested that BMSC-derived exosomes could rescue mitochondrial dysfunction in degenerative chondrocytes. In addition, BMSC-derived exosomes could suppress the activity of osteoclasts in subchondral bone via inhibition of the RANKL-RANK-TRAF6 signaling pathway.^185^

Moreover, BMSC-derived exosomes could also regulate the biological phenotypes of other OA-related cells, including SFB and macrophages.^186^ Jin et al. revealed that human BMSC-derived exosomes inhibited the proliferation and enhanced the apoptosis of IL-1β-treated SFB via a miRNA-26a-5p-mediated decrease in PTGS2.^186^ BMSC-derived exosomes decreased the percentages of F4/80+ macrophages that expressed CD86, MHCII, or CD40 markers. These exosomes also markedly downregulated the level of TNF-α and upregulated that of IL-10, suggesting that BMSC exosomes could inhibit the activation of macrophages in vitro.^182^

In the collagenase-induced murine model, intra-articular injection of BMSC exosomes reduced articular cartilage damage and subchondral bone degradation.^182^ Similarly, the damage to cartilage and subchondral bone in the lumbar facet joint (LFJ) OA model can be largely abrogated by BMSC exosomes.^185^ Moreover, BMSC-derived exosomes could inhibit aberrant nerve invasion and the abnormal formation of H-type vessels in the subchondral bone to relieve pain in LFJ OA mice.^185^ In an experimental rat OA model, hBMSC-derived exosomes decreased synovial tissue proliferation and inflammatory cell infiltration, downregulating the levels of proinflammatory factors and alleviating pathological changes in the synovium.^186^ Most current studies in animal OA models mainly focus on the analysis of pathological changes caused by BMSC-derived exosomes, but there is still a lack of assessments of behavioral changes in these models.

Drug intervention or gene modification could regulate the secretion and contents of exosomes, which may influence the effects of exosomes on their targeted cells.^43,134,187–189^ Mao et al. investigated the influence of exosomes from miR-92a-3p-overexpressing BMSCs on chondrogenesis and cartilage degeneration.^190^ The BMSCs were transfected with miR-92a-3p mimic, and then the exosomes were collected and named MSC-miR-92a-3p-Exos.^190^ They found that MSC-miR-92a-3p-Exos significantly upregulated the levels of aggrecan, SOX9, COL9A1, COL2A1, and COMP and downregulated the expression of COL10A1, RUNX2 and MMP13, suggesting that BMSC-derived exosomes promote chondrogenesis and prevent cartilage matrix degradation in a miR-92a-3p-dependent manner.^190^ Moreover, the MSC-miR-92a-3p-Exos could effectively protect the articular cartilage from damage and delay the progression of early OA in a collagenase-induced mouse model.^190^ In addition, exosomes derived from kartogenin-preconditioned BMSCs were reported to be more effective for cartilage repair and matrix formation in vitro and in vivo than exosomes from BMSCs without kartogenin treatment.^191^ Therefore, the regulation of exosomal secretion and contents by drug intervention or gene modification could be a potential strategy to enhance the therapeutic effectiveness of BMSC-derived exosomes for OA.

##### Exosomes from synovial mesenchymal stem cells

Synovial mesenchymal stem cells (SMSCs) have shown preferable chondrogenic differentiation capacity in vitro.^192–194^ Koizumi et al. also reported that SMSCs from OA or RA patients could efficiently enhance cartilage repair using allogenic tissue engineered constructs in vitro and in vivo.^195^ Intra-articular injection of SMSCs could markedly promote cartilage repair and be used to treat joint-related diseases, including OA, in experimental animal models.^196–199^

Recently, several studies revealed that exosomes derived from SMSCs could effectively promote cartilage regeneration and attenuate OA progression.^122,195,200^ Tao et al. reported that SMSC-derived exosomes could induce the proliferation and emigration of articular chondrocytes in a Wnt5a/Wnt5b/YAP-dependent manner. However, these SMSC exosomes decreased ECM secretion. Exosomes derived from SMSCs transfected with miR-140-5p (SMSC-140-Exos) not only promoted the proliferation and migration of chondrocytes but also prevented ECM damage in vitro.^122^ Moreover, SMSC-140-Exo treatment significantly lessened joint wear, decreased OARSI scores and delayed OA progression in a rat OA model.^122^ Similarly, Zhu et al. found that exosomes isolated from human synovial membrane-derived mesenchymal stem cells (SMMSCs) promoted the migration and proliferation of chondrocytes in vitro.^200^ Moreover, SMMSC exosomes (SMMSC-Exos) elevated ICRS scores, decreased OARSI scores and maintained the collagen II content in a collagenase-induced mouse OA model.^200^ In addition to cartilage homeostasis, exosomes derived from SMMSCs can also regulate bone remodeling in vitro and in vivo.^201^ It was reported that SMMSC-derived exosomes decreased glucocorticoid (GC)-induced fatty cell accumulation, bone marrow necrosis, and trabecular bone loss.^201^ Micro-CT analysis also revealed that SMMSC-Exos significantly improved the microstructures of trabecular bone and mineral density in GC-induced ONFH (femoral head osteonecrosis) in rats.^201^ In addition, SMMSC-derived exosomes partially reversed GC-induced proliferation arrest and the apoptosis of BMSCs in vitro.^201^ As bone remodeling, including subchondral bone change and osteophyte formation, is commonly observed during the OA process and is closely related to OA progression, the effects of exosomes derived from SMMSCs or other cells on bone remodeling in OA pathogenesis are worth investigating.

##### Exosomes from adipose tissue mesenchymal stem cells

Adipose tissue mesenchymal stem cells (AMSCs) were demonstrated to have potent capability for cartilage regeneration and inflammatory modulation and are considered an excellent cell source for OA treatment.^202–207^ However, the mechanisms of AMSC-induced cartilage regeneration are still not clear. Increasing evidence suggests that AMSCs may prevent cartilage erosion and improve joint function primarily through the paracrine secretion of trophic factors to regulate the local microenvironment, making it more favorable for repair and regeneration.^205^ Tofino-Vian et al. reported that EVs, including MVs and exosomes, mainly mediate the paracrine effects of AMSCs on osteoarthritic osteoblasts.^208^ The MVs and exosomes from human AMSCs significantly decreased the activity of senescence-associated β-galactosidase and γH2AX foci accumulation, reduced IL-6 and PGE2 levels, enhanced the release of IL-10, and downregulated mitochondrial membrane potential in IL-1β-treated osteoblasts.^208^ Moreover, AMSC-derived MVs and exosomes could inhibit the production of proinflammatory mediators, such as TNF-α, IL-6, PGE2, and NO, and MMP activity and MMP-13 levels while increasing the levels of the anti-inflammatory cytokine IL-10 and chondrocyte-specific molecule collagen II in OA chondrocytes,^209^ which suggested potential anti-inflammatory and chondroprotective effects of AMSC-derived MVs and exosomes.^209^ These findings provide a new perspective for developing therapeutic approaches for OA based on AMSC-derived EVs.

Recently, our group isolated and identified IPFP MSC-derived exosomes.^146^ We found that MSC(IPFP)-Exos could significantly ameliorate the pathological severity of articular cartilage and partially improve the abnormal gait in DMM-induced OA mice by regulating cartilage homeostasis.^146^ miR-100-5p was abundant in MSC(IPFP)-Exos and may mediate the inhibition of mTOR and autophagy enhancement by binding to the 3′-untranslated region of mTOR.^146^ Similarly, Woo et al. reported that EVs (mean diameter of 86.46 nm) from human adipose-derived stem cells (hASCs) enhanced the proliferation and migration of human OA chondrocytes and maintained the chondrocyte matrix.^210^ Intra-articular injection of hASC-EVs could delay OA progression and protect cartilage from degeneration in both MIA rat and DMM mouse models.^210^ In addition, Zhao et al. isolated ADSCs from donor adipose tissue after elective liposuction surgery and collected exosomes from these cells (ADSCs-Exos).^211^ They found that ADSCs-Exos could downregulate the levels of proinflammatory genes and upregulate the expression of anti-inflammatory cytokines in activated SFB, enhancing the proliferation and chondrogenic potential of periosteal cells via upregulation of miR145 and miR221.^211^ Collectively, these studies further support AMSC-derived exosomes as a potential therapeutic solution for OA in the future.

##### Exosomes from embryonic mesenchymal stem cells

Embryonic MSCs have been regarded as another potential candidate for cartilage regeneration and OA treatment.^212–216^ Recently, some studies revealed that exosomes from embryonic MSCs evidently regulated the biological phenotypes of chondrocytes and delayed OA progression in experimental OA models.^217–221^ In 2016, Zhang et al. successfully isolated and identified exosomes from HuES9 human embryonic stem cells (hESCs).^217^ After intra-articular injections of hESC-derived exosomes into osteochondral defects in rats, they observed that the damage to cartilage and subchondral bone was largely reversed at 6 weeks and almost completely restored at 12 weeks, indicating that embryonic MSC exosomes (MSC-Exos) could be a cell-free therapeutic alternative for cartilage repair and joint-related disease.^217^ Similarly, exosomes from deathless E1-MYC 16.3 human embryonic stem cell-derived MSCs enhanced integration and surface regularity with neighboring host cartilage at 12 weeks and 6 weeks in a rat osteochondral defect model.^218^ Moreover, these exosomes significantly promoted neotissue formation and extracellular matrix deposition, increased M2 macrophage infiltration, and reduced M1 macrophage and proinflammatory cytokine production as early as 2 weeks.^218^ This study also demonstrated that embryonic MSC-derived exosomes could be endocytosed by chondrocytes to regulate chondrocyte migration, proliferation and matrix synthesis through adenosine-dependent AKT and ERK signaling pathways.^218^ In the DMM-induced OA model, exosomes from human embryonic stem cell-induced mesenchymal stem cells (ESC-MSCs) significantly prevented cartilage destruction and matrix degradation after 4 weeks of intra-articular injection.^219^ In addition, exosomes from embryonic MSCs could reduce the inflammatory response, relieve early pain, and promote the repair of cartilage and healing of subchondral bone in an OA model of the temporomandibular joint of immunocompetent rats.^220^ Embryonic MSC-derived exosomes reversed the IL-1β-mediated inhibition of s-GAG synthesis and reduced the IL-1β-induced production of nitric oxide and MMP13 via adenosine-mediated activation of AKT, ERK, and AMPK.^220^ Furthermore, TGF-β1 enhanced miR-135b expression in embryonic MSC-derived exosomes, which may promote chondrocyte proliferation by decreasing the expression of Sp1 and accelerating cartilage repair in a rat OA model.^221^

##### Other stem cell-derived exosomes in OA

Recently, Zavatti, M. et al. explored the effect of amniotic fluid stem cell (AFSC)-derived exosomes on cartilage repair in an MIA-induced animal OA model.^222^ The AFSC exosomes enhanced pain tolerance and induced an almost complete restoration of hyaline cartilage with good surface regularity.^222^ They also observed that AFSC exosomes inhibit M1 polarization, indicating role of AFSC exosomes in regulating inflammation in OA treatment.^222^ Yan et al. revealed that exosomes from umbilical MSCs (U-MSC-Exos) had potent chondroprotective effects, including stimulating chondrocyte proliferation and migration, increasing matrix synthesis and inhibiting chondrocyte apoptosis.^223^ Intra-articular injection of U-MSC-Exos could significantly promote the repair of cartilage defects in vivo.^223^ Moreover, their data also showed that the U-MSC-Exos produced from 3D culture exerted a stronger effect on cartilage repair than those from conventional 2D culture.^223^ In addition, the exosomes of stem cells from human exfoliated deciduous teeth (SHEDs) have been revealed to inhibit inflammatory reactions and maintain anabolism homeostasis in temporomandibular joint chondrocytes via miR-100-5p-mediated mTOR inhibition.^224^ The exosomes produced by induced pluripotent stem cell-derived MSCs (iMSC-Exos) were isolated using an ultrafiltration method and identified by western blot assay, tunable resistive pulse-sensing analysis and TEM.^200^ iMSC-Exos with a diameter of 50–150 nm significantly promoted chondrocyte migration and proliferation in vitro and had a stronger therapeutic effect on OA collagenase-induced mice than SMMSC-Exos, indicating that iMSC-Exos would be more ideal for future use in OA treatment.^200^

#### The exosomal contents in OA

The exosomal contents in OA are the basis of exosome physiological function in intercellular communication and signal transduction.^28^ The contents, including microRNA, long noncoding RNA, DNA, lipid, and protein, could vary with disease progression and greatly contribute to pathological changes in disease.^64,225^ In addition, the exosomal contents were also closely related to the therapeutic effect of MSC-derived exosomes on the repair and regeneration of injured tissues.^226,227^ To date, increasing work has revealed the important roles and underlying mechanisms of exosomal contents during the OA process, including miRNAs, lncRNAs, and proteins. However, other types of RNA and the lipid composition of exosomes in OA pathology have not been reported and need to be further investigated.

##### Exosomal miRNA in OA

The miRNA content of SF exosomes differed between OA- and non-OA-derived samples.^99^ There is significant gender-specific differential expression of exosome miRNAs in SF from OA patients.^99^ In the male group, 69 miRNAs, such as miR-6878-3p, were downregulated, while 45 miRNAs, including miR-210-5p, were upregulated in exosomes isolated from OA-derived SF. In the female group, 91 miRNAs, including miR26a-5p, miR-146a-5p, and miR-6821-5p, were decreased, while 53 miRNAs, including miR-16-2-3p, were increased.^99^ GO and KEGG analyses revealed that specific exosomal miRNAs from female OA SF are closely associated with the estrogen response and TLR (toll-like receptor) signaling pathways.^99^ Mao et al. detected the expression profiles of miRNAs in exosomes derived from OA and normal chondrocytes by miRNA microarray.^109^ They found that the expression of 22 miRNAs was markedly increased, while the expression of 29 other miRNAs was decreased in OA chondrocyte-secreted exosomes in comparison with that in normal chondrocytes.^109^ Compared with normal chondrocytes, exosomal miR-92a-3p^190^ and miR-95-5p^109^ secreted from OA chondrocytes were significantly downregulated. miR-146a-5p was found to be enriched in the EVs of adipose-derived MSCs (ASCs) cultured under inflammatory OA SF conditions and may contribute to OA progression.^228^ In addition, IL-1β treatment could change the miRNA profile of exosomes from human SFB, including the upregulation of 11 miRNAs and downregulation of 39 miRNAs.^121^ Using high-throughput sequencing, 124 differentially expressed miRNAs were identified by screening in OA patient-derived subchondral osteoblasts. Among these miRNAs, hsa-miR-4717-5p had the maximum fold change and targeted a multifunctional regulator of G-protein signaling RGS2.^135^ These studies indicate that exosomal miRNAs from different joint tissues vary with OA disease and are potential candidates for diagnostic OA markers and therapeutic targets.

Exosomal miRNAs play an important role in the therapeutic effects of exosomes on OA (Table 3). Jin et al. reported that miRNAs could be successfully transferred from MSCs to OA-related cells, such as SFB, through exosomes.^186^ The overexpression of miR-26a-5p could regulate hBMSC-exosome function to promote the survival of SFB via downregulation of PTGS2 in vitro and reduce synovitis in a rat OA model.^186^ TGF-β1 could increase the level of miR-135b in MSC-Exos; these exosomes thus showed enhanced stimulation of chondrocyte proliferation in vitro by negatively regulating Sp1 expression and may subsequently promote cartilage repair in vivo.^221^ The exosomes from anti-miR-92a-3p-transfected MSCs inhibited chondrogenic differentiation and the synthesis of cartilage matrix by upregulating the level of WNT5A, while the exosomes from miR-92a-3p-transfected MSCs significantly promoted cell proliferation and increased matrix synthesis in primary OA human chondrocytes.^190^ The exosomes produced by IPFP-MSCs contained abundant miR-100-5p that can bind to the 3′-untranslated region (3′UTR) of mTOR to decrease its expression.^146^ The decreased mTOR may initiate autophagy and restore cartilage homeostasis in vitro and vivo.^146,229–231^ Similarly, miR-100-5p was also enriched in the exosomes of stem cells isolated from human exfoliated deciduous teeth and participated in the suppression of inflammation in TMJ OA chondrocytes via directly targeting mTOR signaling.^224^ The exosomes from miR-140-5p-overexpressing SMSCs (SMSC-140-Exos) could activate the YAP signal and promote the proliferation and migration of chondrocytes without decreasing ECM secretion. These SMSC-140-Exo-mediated effects were mainly dependent on the miR-140-5p/RalA/SOX9 signaling pathway.^122^ The exosomes from normal chondrocytes transfected with miR-95-3p mimicked binding to the 3ʹ-UTRs of HDAC2/8, and maintained cartilage development and homeostasis via the miR-95-3p-mediated transcriptional inhibition of HDAC2/8.^109^ In addition to its protective roles, the miRNA also contributes to the effects of exosomes on joint inflammation and OA progression. The exosomes from osteoarthritic chondrocytes (OC-Exos) could stimulate IL-1beta processing and production in macrophages via miR-449a-5p-mediated autophagy inhibition.^107^ miR-449a-5p can bind to the 3′-UTR of the ATG4B gene to inhibit its expression, which leads to autophagy inhibition and inflammasome activation of macrophages in vitro and may ultimately promote synovitis and cartilage erosion in DMM-induced mice.^107^ Antagonizing miR-449a-5p reversed OC-Exo-mediated proinflammatory effects and cartilage destruction, which provided a new perspective for OA treatment based on miR-449a-5p of OC-Exos.^107^

**Table 3:** The exosomal miRNAs in OA

Exosomes miRNAs	Exosomes source	Target gene	Biological effects	Reference
miR-92a-3p	BMSCs	WNT5A	Promote cell proliferation and increase matrix synthesis in OA primary chondrocytes.	^190^
miR-26a-5p	BMSCs	PTGS2	Promote the survival of synovial fibroblasts and reduce synovitis in Rat OA model.	^186^
miR-135b	EMSCs	Sp1	Enhance chondrocyte proliferation in vitro and promote cartilage repair in vivo.	^221^
miR-100-5p	AMSCsSHEDs	mTOR	Initiate autophagy and restore cartilage anabolism, inhibit catabolism in chondrocytes. Prevent cartilage erosion and suppression of inflammation in experimental OA model.	^146,224^
miR-140-5p	SMSCs	RalA	Increase the expression of SOX9 and Aggrecan of chondrocytes and maintain ECM secretion.	^122^
miR-95-5p	Chondrocytes	HDAC2/8	Increase the level of acetylated histone H3, and maintain cartilage development and homeostasis in vitro.	^109^
miR-449a-5p	Chondrocytes	ATG4B	Inhibit autophagy inhibition and activate inflammasome in macrophages. Promote cartilage erosion and synovitis in DMM-induced OA mice.	^107^
miR-8485	Chondrocytes	GSK3β	Induce chondrogenic differentiation of BMSCs via GSK-3β-mediated Wnt/β-catenin pathway.	^111^

##### The exosomal lncRNAs in OA

Long noncoding RNAs (lncRNAs) are important exosomal contents that participate extensively in the regulation of a wide range of pathological and physiological processes.^232^ Exosomal lncRNAs, such as lncRNA CRNDE-h and lncRNA-p21, are considered possible biomarkers for carcinoma diagnosis and prognosis.^233,234^ Recently, Zhao et al. harvested SF samples from prearthritic patients, early-stage OA patients and late-stage OA patients.^101^ The exosomes from these samples were extracted by ultracentrifugation and subjected to RNA detection.^101^ They found that the exosomal lncRNA PCGEM1 was gradually elevated with OA progression and exhibited a positive relationship with the WOMAC Index (the Western Ontario and McMaster Universities Osteoarthritis Index), suggesting that exosomal lncRNA PCGEM1 could be a potent indicator for OA diagnosis and recognizing the difference between early OA and late OA in clinical practice.^101^ In addition, exosomes can modulate multiple cell phenotypes, including tissue repair and regeneration, via exosomal lncRNAs.^235–238^ Liu et al. reported that MSC-Exos upregulated Col2a1 and aggrecan levels, downregulated MMP13 and Rux2 expression, and promoted the survival of IL-1β-treated chondrocytes mainly through exosomal lncRNA-KLF3-AS1.^239^ The lncRNA KLF3-AS1 enhanced the expression of GIT1 by sponging miR-206 in vitro.^240^ The overexpression of miR-206 or knockdown of GIT1 partially attenuated MSC(KLF3-AS1)-Exo-mediated effects on chondrocyte repair.^240^ These studies suggested that exosomal lncRNA-KLF3-AS1 plays an important role in the effect of MSC-Exos on osteoarthritic chondrocytes mainly through targeting the miR-206/GIT1 axis.^240^ Moreover, exosomal lncRNA-KLF3-AS1 significantly contributed to MSC-exo-mediated cartilage repair in a collagenase II-induced OA model.^239^

##### The exosomal proteins in OA

Several types of specific cellular proteins can be selectively sorted into MVBs and secreted as exosome loading cargo.^241–243^ Exosomal proteins have been demonstrated to contribute to cell–cell communication and signal transduction, which are highly involved in various diseases.^244–246^ The synovial exosomes from RA patients contained potential autoantigenic content, including citrullinated proteins, which may participate in the autoimmune reaction.^97^ Tsuno et al. extracted serum exosomal proteins from the healthy group, OA group and RA group and identified the exosomal proteins via 2D-DIGE and mass spectrometry methods.^247^ They found that the exosomal protein profiles from the serum of the three groups were significantly different. There were 21 spots in the exosomal protein profiles with different intensities between the OA group and the healthy group, such as cathepsin F and Igalpha-2 chain C region, indicating the potential roles of these proteins in OA.^247^ Moreover, exosomal proteins have been revealed to participate in the exosome-mediated regulation of the biological response in chondrocytes. CD73/ecto-5′-nucleotidase was expressed in MSC-Exos. The CD73 inhibitor AMPCP or the nonselective adenosine receptor antagonist theophylline could reduce MSC exosome-induced AKT and ERK phosphorylation in chondrocytes, indicating that exosomal CD73/ecto-5′-nucleotidase played an important role in the biological effect of MSC-Exos on cartilage.^218^

## Perspective

Exosomes may become potent candidate risk factors and early diagnostic markers for clinical OA patients in the future. Subgroups of exosomes distinguished by the levels or activity of exosome-specific proteins were highly associated with cancer onset and progression and can be used to detect early cancer.^63,248^ In addition, miRNAs and lncRNAs in exosomes have also been considered as potential diagnostic markers for several kinds of diseases, including RA and OA.^99,101,234,249–251^ More studies are needed to identify the different subgroups of exosomes from OA peripheral blood or SF samples and analyze their relationship with OA pathological changes and clinical classification. In addition, we can attempt to identify the original secreted cells of exosomes based on exosome-specific markers. Moreover, urine can be harvested through noninvasive approaches, which may be more acceptable for patients in clinical practice. Previous studies have revealed that the components of urine were changed with OA progression and could predict cartilage degradation as a biochemical marker, indicating that urine could be used to evaluate joint-related diseases.^252–254^ In addition, the contents from urine exosomes are highly relevant to several types of disorders, including cancer, and could be used as potential diagnostic markers for these diseases.^255–257^ Therefore, exosomes from body fluids other than blood, such as urine, should be given additional attention to explore their availability as diagnostic markers for OA patients.

The endogenous exosomes in our body have many physiological and pathological functions and have been considered potent therapeutic targets in a variety of diseases.^258–260^ The roles and underlying mechanisms of exosomes from different joint tissues in OA pathogenesis are quite variable and could be affected by many factors, including sex, obesity, aging, basic diseases, therapeutic intervention, joint motion, and microenvironment. For example, exosomes from chondrocytes cultured in a normal environment can increase M2 macrophage infiltration and delay OA progression,^106^ while exosomes from IL-1β-treated chondrocytes promote inflammasome activation in macrophages and aggravate OA pathological severity.^107^ Intensive studies of endogenous exosomes originating from joint cells during the OA process would be beneficial for developing novel targeted therapeutic strategies. More investigations should aim to reduce the release of pro-OA (promoting OA onset and progression) exosomes and the pro-OA contents of these exosomes or inhibit exosome-mediated signal transduction in targeted cells. Conversely, some measures could be taken to increase the production of endogenous anti-OA (inhibiting OA onset and progression) exosomes, such as IPFP MSC-Exos and synovial MSC-Exos,^146,197^ and to enhance their anti-OA capacity by acting on the target cells.

To date, several types of exosomes derived from MSCs of different tissues have exhibited protective effects on cartilage in vitro and in vivo. However, the separation methods for these MSC-derived exosomes are quite different (Table 3) and have some disadvantages, which may influence clinical standardization and potential application. Ultracentrifugation, the most classic separation method, usually requires extended time to harvest exosomes in vitro, which may increase the risk of infection. PEG-mediated isolation of exosomes may influence the biological activity of exosomes for subsequent clinical application. Chen et al. compared the efficiency of three separation techniques (ultracentrifugation, filtration combined with size exclusion chromatography and 8% polyethylene glycol) for extracting synovial tissue-derived exosomes (Syn-Exos).^261^ They found that the efficiency of exosome isolation differed among these three isolation methods and recommended ultracentrifugation and filtration combined with size exclusion chromatography for the extraction of Syn-Exos.^261^ Therefore, additional measures are needed to optimize and standardize the existing separation methods so that the clinical use of MSC-Exos is safe and convenient.

Regulation of the secretion and contents of exosomes by drug intervention or gene/material modification contributes greatly to the therapeutic action of MSC-Exos in diverse diseases,^262–265^ so it is advisable to develop engineering technology for MSC-Exos for OA treatment. Recently, MSC-Exos combined with a hydrogel sponge were used to accelerate wound healing and regeneration in animal injury models, including a cartilage damage model,^266–269^ suggesting a potential application of this technology in OA treatment. The hydrogel sponge could make the MSC-Exos more stable in vivo and might better control the release of MSC-Exos according to changes in the joint cavity microenvironment, such as increased proinflammatory factors and matrix metalloproteinase activity or excessive mechanical load. Apart from MSCs, exosomes from other cells or tissues may also have a potential therapeutic effect on OA. A recent study revealed that exosomes derived from platelet-rich plasma (PRP-Exos) can significantly protect cartilage from damage by activating the Wnt/β-catenin signaling pathway, whose therapeutic effect was even better than that of activated PRP.^270^ Additional research is needed to improve the therapeutic effects of exosomes derived from different sources.

Nevertheless, there are also some challenges and problems in this field. There is still a lack of direct evidence that endogenous exosomes could transfer from one cell to another cell in the joint in vivo, which restricts further studies, such as the identification of the major target cells of different exosomes. Moreover, the mechanisms of exosome generation and release in the joint are still unclear, limiting exosome-based targeted intervention. In addition, there are some difficulties in achieving the protective effects of MSC-Exos on chondrocytes during the early stage of OA. As cartilage is relatively complete at the early OA stage, it may be difficult for exosomes to permeate through the cartilage matrix to enter chondrocytes, especially the chondrocytes in the deep layer. Therefore, the key points of engineering MSC-Exos for use in the early OA stage may focus on superficial chondrocytes, synovial cells, and other joint cells that are easily accessible by exosomes, or on cartilage matrix maintenance.

## Conclusions

As an important intercellular communication mediator, exosomes greatly contribute to OA onset and progression and have shown strong potential for OA treatment. Exploring the detailed mechanisms of exosomes in OA pathological changes will help us to screen and identify potential therapeutic targets. Moreover, we need to optimize MSC-Exos to improve their therapeutic effects on OA. Studies on the roles, underlying mechanisms, and diagnostic/therapeutic application of exosomes in OA are only beginning, and there are still many problems to be solved in this field. With advances in technology, we speculate that exosome-based treatment will be applied to OA patients in the future.
